# The association between *BRAF* mutation class and clinical features in *BRAF*-mutant Chinese non-small cell lung cancer patients

**DOI:** 10.1186/s12967-019-2036-7

**Published:** 2019-08-30

**Authors:** Quan Lin, Haoran Zhang, Huaxin Ding, Jun Qian, Analyn Lizaso, Jing Lin, Han Han-Zhang, Jianxing Xiang, Yuping Li, Hong Zhu

**Affiliations:** 1 Department of Pulmonary and Critical Care Medicine, The First Affiliated Hospital of Wenzhou Medical University, Nanbaixiang Campus, Ouhai District, Wenzhou, 325015 Zhejiang China; 2 Department of Medical Oncology, The First Affiliated Hospital of Bengbu Medical College, Bengbu, 233000 Anhui China; 3 Ningbo Diagnostic Pathology Center, Ningbo, 315000 Zhejiang China; 4 Department of Oncology, The Affiliated Suzhou Hospital of Nanjing Medical University, Suzhou, 215001 Jiangsu China; 5 Burning Rock Biotech, Guangzhou, 510300 Guangdong China; 6 Department of Oncology, The First Affiliated Hospital of Soochow University, No. 899 Pinghai Road, Gusu District, Suzhou, 215006 Jiangsu China

**Keywords:** BRAF, Non-small cell lung cancer, Chinese

## Abstract

**Background:**

*BRAF* mutations occur in 2–4% non-small cell lung cancer (NSCLC) patients and can be categorized into three functional classes based on signaling mechanism and kinase activity: RAS-independent kinase-activating V600 monomers (class 1), RAS-independent kinase-activating dimers (class 2) and RAS-dependent kinase-inactivating heterodimers (class 3). The association between functional classes and clinical features in Chinese NSCLC patients remains unexplored. Our multi-center study aimed to survey the *BRAF* mutation rate and analyze the associated clinical features in this population.

**Methods:**

Capture-based sequencing data of either plasma or tissue samples obtained from 8405 Chinese stage I–IV NSCLC patients were retrospectively analyzed.

**Results:**

*BRAF* mutations were detected in 238 patients, revealing an overall mutation rate of 2.8%. Among them, 32%, 21% and 13% had *BRAF* mutant class 1, 2 and 3 respectively. The remaining 34% had other *BRAF* mutations. V600 (32%) and G469 (13%) were the two most predominant *BRAF* mutations. Patients with class 2 and 3 mutations were more likely to have concurrent *KRAS* mutations (*P *= 0.001). Collectively, *BRAF* mutations, including non-class 1–3 mutations, were more likely to occur in males (*P *< 0.01). However, females were more likely to harbor class 1 mutations (*P *< 0.02). We also compared the overall survival (OS) of first-line chemotherapy-treated advanced-stage patients and revealed comparable OS among the three groups.

**Conclusion:**

Our study revealed a 2.8% *BRAF* mutation rate in Chinese NSCLC patients. Our data also showed a male predominance when all *BRAF* mutations were considered collectively, and a female predominance for class 1 mutations. Furthermore, BRAF V600E is less likely to have concurrent *KRAS* mutations comparing to the other two classes.

## Background

The discovery of oncogenic drivers has revolutionized the therapeutic management of cancer patients to a more personalized approach based on the genomic alterations detected in the patient’s tumor. Genomic studies on non-small cell lung cancer (NSCLC) have identified B-Raf proto-oncogene (*BRAF*) as one of the major oncogenic drivers, occurring in 2–4% NSCLC patients [1, 2]. Mutations in *BRAF*, a cytosolic serine/threonine kinase downstream of the Kirsten rat sarcoma oncogene (KRAS), result in the constitutive activation of the mitogen-activated protein kinase (MAPK) signaling pathway, promoting cell growth and proliferation [3–5]. A vast majority of *BRAF* mutations are localized in the kinase domain, including the most commonly observed V600E mutation [6]. In addition to V600E, other non-V600E mutations with distinct kinase activity have also been reported [6]. Based on the mechanism of activation, kinase activity, and sensitivity to inhibitors, a functional mutation classification system has been recently introduced. According to functional class, RAS-independent kinase-activating V600 monomers are categorized as class 1; RAS-independent kinase-activating dimers that are resistant to vemurafenib are categorized as class 2; and RAS-dependent kinase-inactivating heterodimers are categorized as class 3 [6, 7]. Studies have shown that advanced NSCLC patients with class 1 V600E mutations have unfavorable prognosis with first-line chemotherapy relative to *BRAF* wild-type patients [8, 9]. *BRAF* inhibitor monotherapy or in combination with a MEK inhibitor, significantly improves their survival outcomes [10–14]. Studies on V600E-mutant NSCLC patients demonstrated an overall response rate (ORR) of 42% and a median progression-free survival (PFS) of 7.3 months for vemurafenib used as a single agent [11] and an ORR of 33% and PFS of 5.5 months for dabrafenib used as monotherapy [12]. Other studies have evaluated the efficacy of combinatorial treatment, consisting of a BRAF inhibitor, dabrafenib and a MEK inhibitor, trametinib and reported an ORR of 63% and PFS of 9.7 months [13, 14]. On the contrary, the prognosis of patients with non-V600 class 2 and 3 mutations remains controversial, with some reports demonstrating a trend of better prognosis [9, 15] and others showing a trend of less favorable prognosis [16, 17] but some of these findings did not reach statistically significant difference compared with V600E-mutant patients [9, 15, 17]. Meanwhile, some studies have also demonstrated that patients with non-V600 mutations to have comparable prognosis with *BRAF* wild-type patients [8].

Numerous reports have elucidated the prevalence, distribution and prognosis of Chinese *BRAF*-mutant NSCLC patients; however, most of these studies focused on V600E with limited number of patients [18, 19]. In addition, most of the studies have employed traditional molecular testing methods which restricted the discovery of non-V600E mutations [15, 18–20]. In our present multi-center study, we retrospectively analyzed the next-generation sequencing data of 8405 Chinese NSCLC patients from 5 cancer centers to survey the prevalence of *BRAF* mutations, to investigate the distribution of *BRAF* mutations according to the new functional classification system, and to analyze the association between functional class and clinical features in this population.

## Patients and methods

### Patient data

Targeted sequencing results obtained from 4407 plasma and 3998 tissue samples of NSCLC patients who underwent comprehensive molecular testing at Burning Rock Biotech between May 2015 to October 2018 were retrospectively screened for *BRAF* mutations. Medical records from the *BRAF*-mutant patients were retrieved to gather clinicopathologic data, treatment history and survival outcome. This study has been approved by the relevant Institutional Review Board of all the participating hospitals. Written informed consent was provided by all the patients included in the study.

### Tissue and cell-free DNA isolation

Tissue DNA was extracted from formalin-fixed, paraffin-embedded (FFPE) tumor tissues using QIAamp DNA FFPE tissue kit (Qiagen). Likewise, circulating cell-free DNA (cfDNA) was recovered from 4 to 5 ml of plasma using the QIAamp Circulating Nucleic Acid kit (Qiagen).

### Capture-based targeted DNA sequencing

A minimum of 50 ng of DNA is required for NGS library construction. Tissue DNA was sheared using Covaris M220 (Covaris, MA, USA), followed by end repair, phosphorylation and adaptor ligation. Fragments between 200 and 400 bp from the cfDNA and sheared tissue DNA were purified (Agencourt AMPure XP Kit, Beckman Coulter, CA, USA), followed by hybridization with capture probes baits, hybrid selection with magnetic beads and PCR amplification. The quality and the size of the fragments were assessed using Qubit 2.0 fluorimeter with the dsDNA high-sensitivity assay kit (Life Technologies, Carlsbad, CA). Indexed samples were sequenced on Nextseq 500 (Illumina, Inc., USA) with paired-end reads and average sequencing depth of 1,000X and 10,000X for tissue and plasma samples, respectively. Panels from Burning Rock Biotech including 8 lung cancer actionable genes (Lung Cure), 68 lung cancer-related genes (Lung Core), 168 genes including 68 lung cancer-related genes and 100 other genes related to cancer development (Lung Plasma) or 295 cancer-related genes (OncoScreen) were used for targeted sequencing.

### Sequence data analysis

Sequence data were mapped to the reference human genome (hg19) using Burrows-Wheeler Aligner v.0.7.10. Local alignment optimization and variant calling were performed using Genome Analysis Tool Kit v.3.2 and VarScan. Variants were filtered using the VarScan fpfilter pipeline, loci with depth less than 100 were filtered out. Base calling in plasma and tissue samples required at least 8 supporting reads for single nucleotide variations (SNV) and 2 and 5 supporting reads for insertion-deletion variations (INDEL), respectively. Variants with population frequency over 0.1% in the ExAC, 1000 Genomes, dbSNP or ESP6500SI-V2 databases were grouped as single nucleotide polymorphisms (SNP) and excluded from further analysis. Remaining variants were annotated with ANNOVAR and SnpEff v.3.6. Analysis of DNA translocation was performed using Factera v.1.4.3. Copy number variations (CNV) were analyzed based on the depth of coverage data of capture intervals. Coverage data were corrected against sequencing bias resulting from GC content and probe design. The average coverage of all captured regions was used to normalize the coverage of different samples to comparable scales. Copy number was calculated based on the ratio between the depth of coverage in tumor samples and average coverage of an adequate number (n > 50) of samples without copy number variation as references as to each capture interval. CNV is called if the coverage data of the gene region was quantitatively and statistically significant from its reference control. The limit of detection for CNVs is 1.5 and 2.64 for deletions and amplifications, respectively.

### BRAF mutation classification

*BRAF* mutations were classified based on their functional class according to the new classification system and summarized in Table 1 [6, 7].

**Table 1:** *BRAF* mutations included in each functional class

	BRAF mutations
Class 1	V600E/L
Class 2	L597Q/R, G464V/A, G469A/V/R/S, K601E/N/T, E451Q, A712T, fusions
Class 3	G469E, G466V/E/A, N581S/I, D594G/N, G596R

### Statistical analysis

Differences in the groups were calculated and presented using either Fisher’s exact test or paired, two-tailed Student’s *t* test, as appropriate. Associations of *BRAF* mutation status with clinical features were analyzed using univariate logistic regression analysis. Binomial proportion was used to analyze the gender distribution within the mutation class. Overall survival was defined from the date of diagnosis until the day of death or last day of follow-up. Overall survival curve was estimated using Kaplan–Meier method and the differences among the groups were evaluated using the log-rank test. *P*-value with *P *< 0.05 was considered as statistically significant. All the data were analyzed using R statistics package (R version 3.4.0; R: The R-Project for Statistical Computing, Vienna, Austria).

## Results

### Patient characteristics

To survey the prevalence of *BRAF* mutations in Chinese NSCLC patients, 8405 patients who underwent comprehensive molecular testing using capture-based targeted next-generation sequencing were screened. The screened population consisted of 56% (4707/8405) males and 44% (3698/8405) females, with a median age of 61 years.

Among the screened population, *BRAF* mutations were detected in 238 patients. Of the *BRAF*-mutant patients, 65.5% (156/238) were males and 33.6% (80/238) were females, revealing a male predominance (*P *< 0.01). The median age was 61 years, ranging from 33 to 86 years. A majority was diagnosed with adenocarcinoma (79%, 188/238), 11.3% (27/238) had adenosquamous carcinoma, 7.6% (18/238) had squamous cell carcinoma, and 2.1% (5/238) had large cell carcinoma. Thirty-one percent (31%, 74/238) were stage I-IIIA and 69% (164/238) were stage IIIB-IV. A total of 9 V600E mutant patients were administered with BRAF inhibitors, including vemurafenib (n = 7), dabrafenib (n = 1) and combination therapy of dabrafenib and trametinib (n = 1). Twenty-eight patients (11.8%, 28/238) with concurrent sensitizing *EGFR* mutations received EGFR inhibitors. Among them 3 were V600E mutant, 7 were G469X mutants, 3 were G466X mutants, 1 was N581S mutant and the remaining had other *BRAF* mutations. The remaining 183 patients, including 62 V600E, 1 V600L and 136 non-V600E-mutant patients received chemotherapy either as first-line therapy or adjuvant therapy. The clinical and pathological features of the *BRAF*-mutant patients were summarized in Table 2.

**Table 2:** Clinicopathologic characteristics of the 238 *BRAF*-mutant NSCLC patients

	Total (n = 238)	Class 1 (n = 75)	Class 2 (n = 51)	Class 3 (n = 32)	Non-class 1–3 (n = 80)	*P*-value (1 vs. 2)	*P*-value (1 vs. 3)	*P*-value (2 vs. 3)
Age						0.87	0.70	0.75
Median (range)	61 (33–86)	61 (42–82)	61 (45–81)	62 (47–81)	59 (33–86)			
Gender						*0.008*	*0.017*	1
Male	156 (65.5%)	38 (50.7%)	39 (76.5%)	25 (78.1%)	54 (67.5%)			
Female	80 (33.6%)	35 (46.7%)	12 (23.5%)	7 (21.9%)	26 (32.5%)			
NA	2 (0.8%)	2 (2.7%)	0 (0%)	0 (0%)	0 (0%)			
Histology						0.74	0.88	0.81
Adenocarcinoma	188 (79.0%)	60 (80.0%)	40 (78.4%)	28 (87.5%)	60 (75.0%)			
Squamous cell carcinoma	18 (7.6%)	2 (2.7%)	3 (5.9%)	1 (3.1%)	12 (15.0%)			
Adenosquamous carcinoma	27 (11.3%)	10 (13.3%)	7 (13.7%)	3 (9.4%)	7 (8.8%)			
Large cell carcinoma	5 (2.1%)	3 (4.0%)	1 (2.0%)	0 (0%)	1 (1.2%)			
Stage						1	1	1
Stage IA–IIIA	67 (28.2%)	20 (26.7%)	14 (27.5%)	9 (28.1%)	24 (30.0%)			
Stage IIIB–IVB	164 (68.9%)	54 (72.0%)	35 (68.6%)	21 (65.6%)	54 (67.5%)			
NA	7 (2.9%)	1 (1.3%)	2 (3.9%)	2 (6.3%)	2 (2.5%)			
Metastasis						0.43	0.63	1
M0	171 (71.8%)	55 (73.3%)	34 (66.7%)	22 (68.8%)	60 (75.0%)			
M1	67 (28.2%)	20 (26.7%)	17 (33.3%)	10 (31.2%)	20 (25.0%)			

*P*-values in italics-face denotes statistical significance

### Prevalence of BRAF mutations and their distribution

**Fig. 1:**
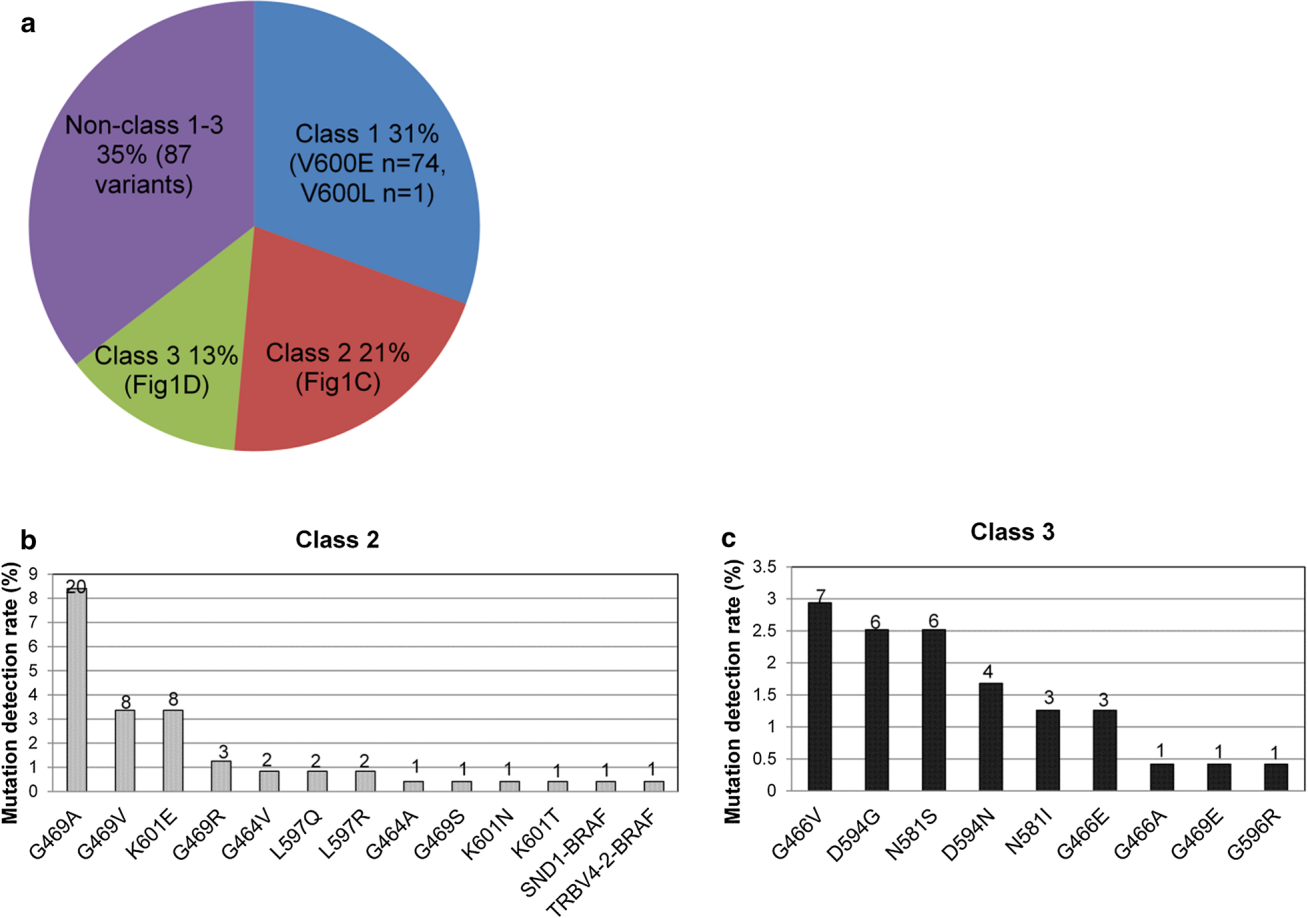
*BRAF* mutation distribution. **a** Distribution of *BRAF*-mutant patients categorized according to class. The detection rate of the mutations in class 2 (**b**) and class 3 (**c**). X-axis denotes the *BRAF* mutations. Y-axis denotes the mutation detection rate. The numbers on the bars indicate the corresponding detection count, or the total number of patients with the specified mutation

Of the 8405 NSCLC patients, a total of 245 *BRAF* mutations were detected in 238 patients, revealing an overall mutation rate of 2.8%. Among them, 31.5% (75/238), 21.4% (51/238), 13.4% (32/238) of the patients had *BRAF* mutant class 1, 2 and 3, respectively. The remaining 33.6% (80/238) of the patients had *BRAF* mutations not classified as class 1–3 (Fig. 1a). All patients with Class 1 V600 mutations had V600E (n = 74) except for 1 patient who had V600L (Fig. 1a and Additional file 1: Figure S1A). The detailed distributions of patients with class 2 or 3 mutations were shown in Fig. 1b, c, respectively. Of the patients with class 2 mutations, G469 (13.1%, 32/245), including G469A (n = 20), G469 V (n = 8), G469R (n = 3) and G469S (n = 1), was the most predominant mutation (Fig. 1b, Additional file 1: Figure S1A). Among the class 3 mutations, G466 (7 G466 V, 3 G466E and 1 G466A) and D594 (6 D594G and 4 D594 N) were the 2 most predominant mutations (Fig. 1c, Additional file 1: Figure S1A).

Collectively, a vast majority of the *BRAF* mutations detected in our cohort were missense mutations (84.5%, 207/245). Other less frequent mutation types included nonsense mutations, small insertion-deletions, splice site variants, frameshifts, fusions and copy number variations (CNVs) (Table 3). In addition to the detection of common and other previously reported mutations, we identified 66 *BRAF* mutations which were not included in the Catalogue of Somatic Mutations in Cancer (COSMIC) variant database. A majority (94%, 63/66) of the novel *BRAF* mutations were non-class 1–3; while the remaining 3 were class 2 mutations. The novel *BRAF* mutations detected in the cohort were summarized in Additional file 2: Table S1 and were depicted with two asterisks (**) in Additional file 1: Figure S1A and B. Furthermore, 7 patients (2.9%, 7/238) had compound *BRAF* mutations. The *BRAF* mutations detected in these 7 patients were summarized in Additional file 2: Table S2. Among these 7 patients, 1 patient had concurrent class 1 *BRAF* V600E and *BRAF* amplification; 2 patients had a class 2 mutation in combination with an “other mutation” (non-class 1–3 mutation); 1 patient had concurrent class 3 (N581S) and non-class 1–3 (D66E) *BRAF* mutations. Both mutations of the remaining 3 patients were non-class 1–3 *BRAF* mutations. Of the patients with compound non-class 1–3 *BRAF* mutations, 2 patients had mutations that were *in cis* including a male patient with L858F *in cis* to L505H (Additional file 1: Figure S2B) and a female patient with BRAF S316L *in cis* to S317C (Additional file 1: Figure S2C) who also had concurrent *EGFR* exon 19 deletion.

**Table 3:** *BRAF* mutation types detected in the cohort

Mutation types	Total	Class 1	Class 2	Class 3	Non-class 1–3
Missense	207	75	49	26	57
Nonsense	5	0	0	0	5
Small insertion deletion (including disruptive indels)	13	0	0	0	13
Splice site	10	0	0	6	4
Frameshift	4	0	0	0	4
Fusion	3	0	2	0	1
Copy number deletion	2	0	0	0	2
Copy number amplification	1	0	0	0	1
Total	245	75	51	32	87

### Concurrent oncogenic driver mutations

**Fig. 2:**
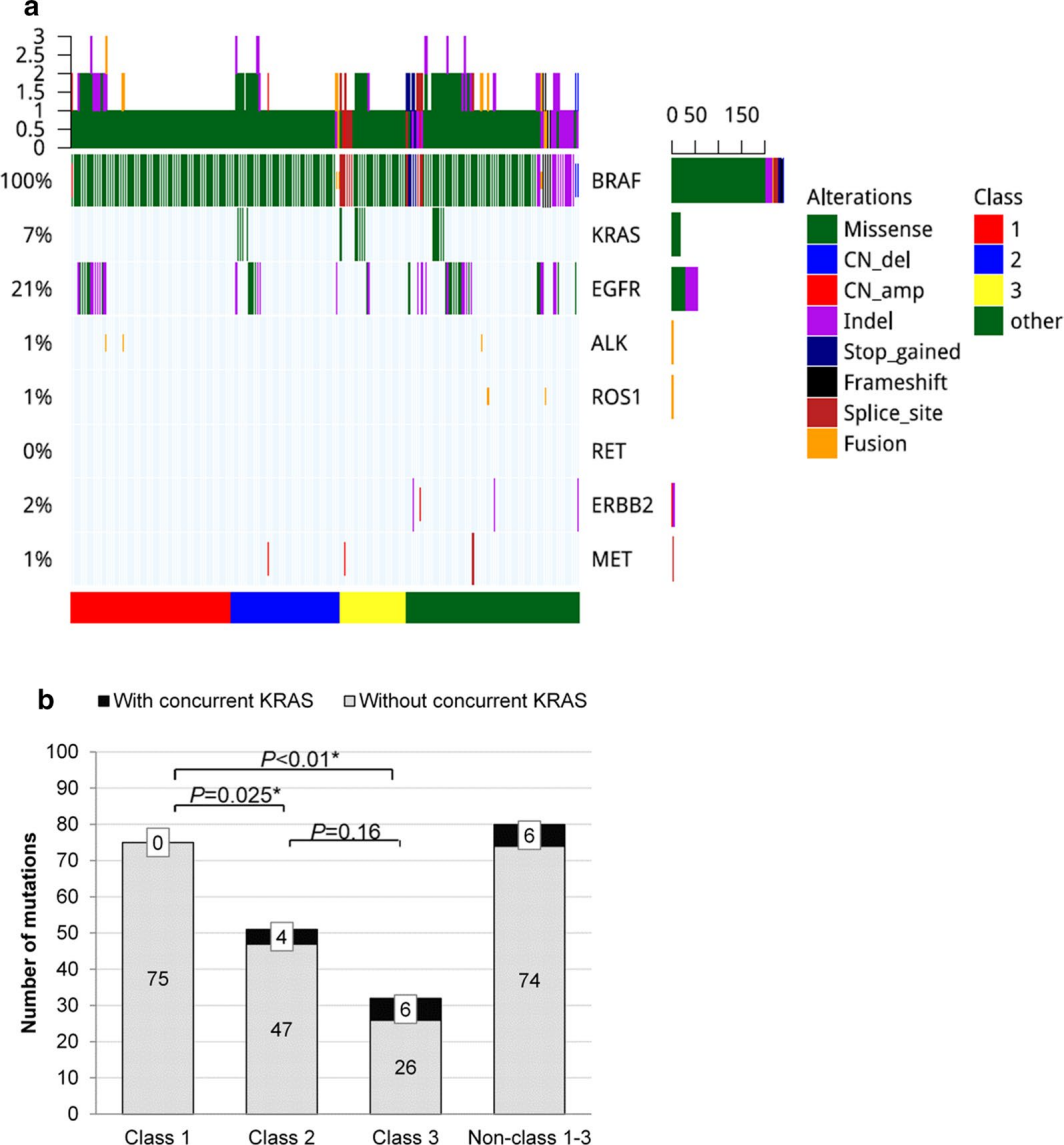
Concurrent oncogenic mutations based on *BRAF* mutation class. **a** Mutation spectrum in the 8 classic NSCLC oncogenic driver of the 238 *BRAF*-mutant patients. The patients were grouped according to *BRAF* mutation class as indicated by the bar located at the bottom of the oncoprint. Each column represents a patient and each row represents a gene. Table on the left represents the mutation rate of each gene. Top plot represents the overall number of mutations a patient carried. Different colors denote different types of mutation. **b** Concurrent *KRAS* mutations in different *BRAF* mutation classes. Class I *BRAF* mutations were mutually exclusive from *KRAS*, while class 2 (*P *= 0.025) and 3 (*P *< 0.01) were more likely to have concurrent *KRAS* mutations. X-axis denotes the *BRAF* mutant class. Y-axis denotes the number of mutations in either *BRAF* or *KRAS*

Next, we investigated classic lung cancer driver mutations that co-occur with *BRAF* mutations in this cohort. Collectively, 76 patients had concurrent NSCLC driver mutations, including 49 with *EGFR*, 16 with *KRAS*, 4 with *ERBB2* amplifications, 3 with *MET* alterations, 3 with *ALK* fusions, and 2 with *ROS1* fusions (Fig. 2a, Additional file 2: Table S3). When all the classic NSCLC driver mutations were considered collectively, there was no correlation between the likelihood of having concurrent oncogenic driver mutations and *BRAF* mutation class (*P *= 0.66). Further univariate analyses revealed that class 1 *BRAF* mutations were mutually exclusive with *KRAS* mutations. In our cohort, none of the 75 patients with class 1 *BRAF* mutation had concurrent *KRAS* mutation. However, 4 patients (7.8%) with class 2 mutations and 6 patients (19.3%) with class 3 mutations had concurrent oncogenic *KRAS* mutation (G12X, G13X and Q61X) (*P *< 0.001, Fig. 2b). Collectively, our data revealed a mutual exclusivity between class 1 *BRAF* mutation and oncogenic *KRAS* mutation, while class 2 and 3 mutations were more likely to have concurrent *KRAS* mutations.

### Association between BRAF mutations and clinical features

**Fig. 3:**
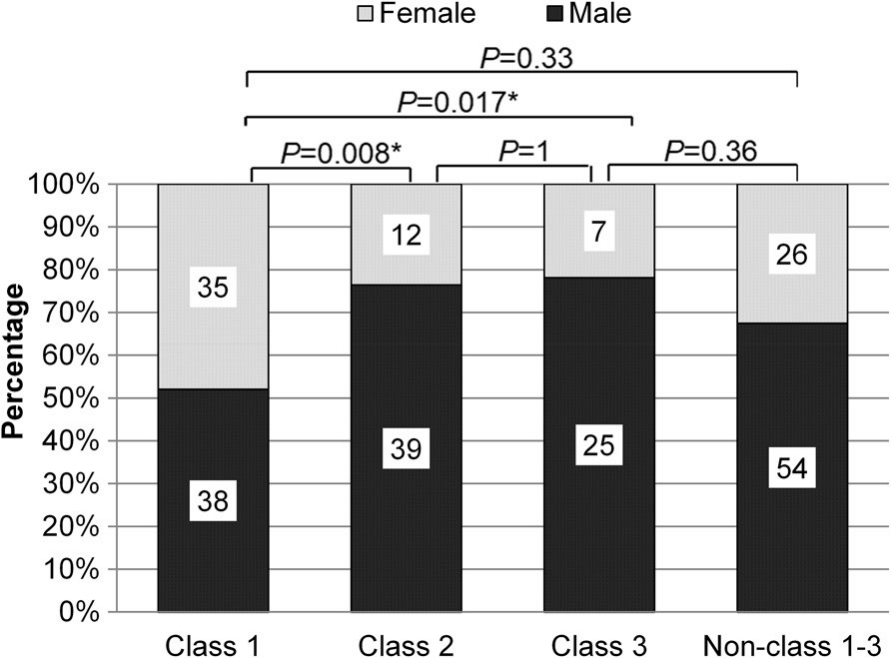
Gender distribution according to *BRA*F mutation class. In general, *BRAF* mutations were more frequently detected among males. However, class I *BRAF* mutations were more predominant in females (*P *= 0.008, *P *= 0.017). X-axis denotes the *BRAF* mutant class. Y-axis denotes the percentage of males or females per mutant class. The numbers indicated are the actual number of males (green) or females (red) per mutant class

We further analyzed the correlation between *BRAF* mutations and clinicopathologic features. Our data revealed that *BRAF* mutations were more likely to occur in males (65.5% vs 33.6%, *P *< 0.01). When gender distribution was analyzed by *BRAF* mutation class using binomial proportion test, both class 2 (76.5% vs. 23.5%, *P *< 0.001) and 3 (78.1% vs. 21.9%, *P *= 0.003) exhibited male predominance; while class 1 did not show any gender preference (50.7% vs. 46.7%, *P *= 1, Table 2, Fig. 3). However, when analyzed collectively, females were more likely to have class 1 mutations than any other *BRAF* mutation class (class 1 vs. 2 *P *= 0.008; 1 vs. 3 *P *= 0.017; Fig. 3). Other clinicopathologic features, including age, histology, stage, and presence of metastasis, were not significantly associated with *BRAF* mutation class.

### Survival outcomes

**Fig. 4:**
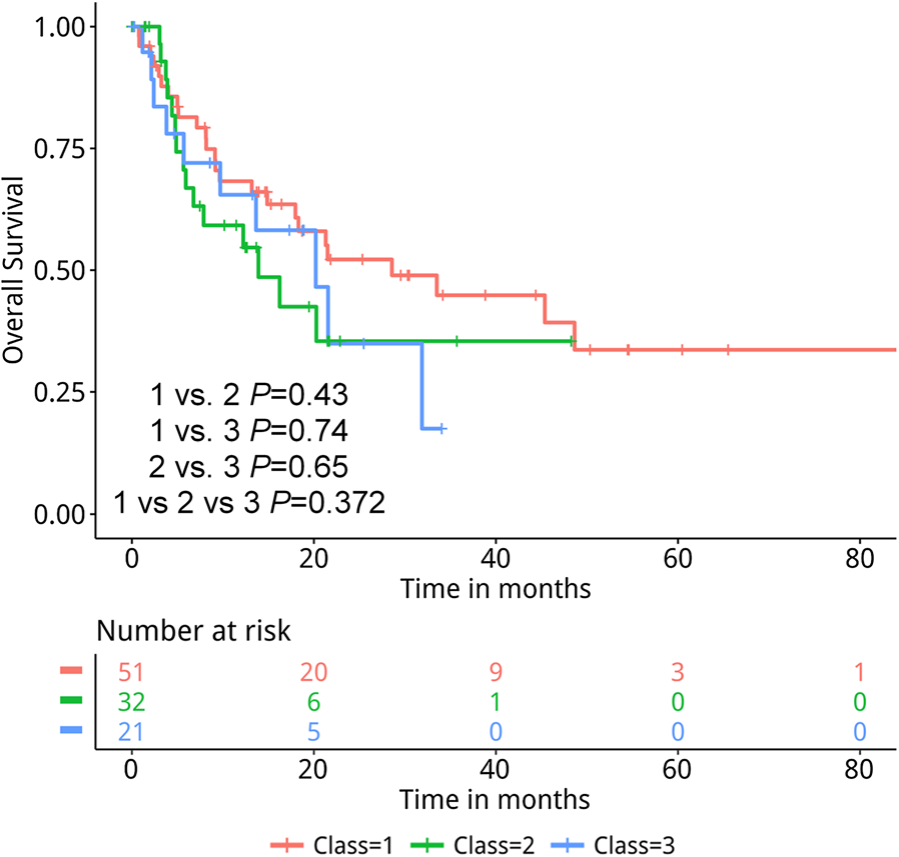
Kaplan–Meier analysis of the overall survival of the 105 *BRAF*-mutant advanced NSCLC patients treated with first-line chemotherapy. The risk table below illustrates the number of patients included per time point

We have also analyzed the survival outcomes based on the *BRAF* mutation class in 105 evaluable stage IIIB-IV patients treated with first-line chemotherapy regimen. Among them, 51, 32 and 21 had class 1, 2 and 3 *BRAF* mutations, respectively. Kaplan–Meier and log-rank analysis revealed comparable overall survival among the three *BRAF* mutation classes, with a median overall survival of 28.6, 13.9 and 20.2 months for class 1, 2 and 3, respectively (*P *= 0.585, Fig. 4).

## Discussion

*BRAF* mutations are clinically significant genetic alterations which occur in 2–4% of NSCLC patients. Despite the poor survival outcome of *BRAF* V600E-mutant NSCLC patients as compared to patients with wild-type *BRAF* [8], treatment with BRAF inhibitors have significantly improved their prognosis. With no approved targeted therapy for non-V600E *BRAF* mutant patients, chemotherapy still remains as the standard treatment option. Efforts to elucidate the prevalence and distribution of *BRAF* mutations according to functional class could facilitate the development of optimal treatment strategies to improve the prognosis of these subsets of patients.

Among Caucasian NSCLC patients, *BRAF* mutations were detected at a frequency of 2–4% [8, 9, 17, 21–23]. Similarly, *BRAF* mutations among the Chinese NSCLC patients ranged from 1.2% (14/1139) to 4.2% (8/190) [15, 18, 19, 24, 25]. In our effort to survey the prevalence of *BRAF* mutations in Chinese NSCLC patients, we have conducted a multi-center retrospective study involving 5 cancer centers. To the best of our knowledge, our study is the largest survey of the prevalence of *BRAF* mutations and the first to interrogate the mutation distribution based on the new functional classification system in Chinese NSCLC patients. We believe that the inclusion of a large cohort in our study reflects the actual prevalence and distribution of *BRAF* mutations in this population.

Among the 8405 stage I–IV NSCLC patients, we have detected *BRAF* mutations in 238 patients revealing an overall *BRAF* mutation rate of 2.8%. The distribution of *BRAF* mutations according to functional class consisted of 32%, 21%, 13% and 34% for class 1, 2, 3 and non-class 1–3, respectively. The mutation distribution in our cohort is consistent with the reported distribution based on the *BRAF* mutation class in non-Asian NSCLC patients [16, 17, 26, 27]. The heterogeneous distribution in our cohort further suggests that only about 30% of the V600E-mutant NSCLC patients can benefit from BRAF inhibitors, while the development of novel therapeutic strategies is crucial to further improve the survival of a majority of *BRAF*-mutant patients. In addition to well-characterized mutations in classes 1 to 3, we have also detected 66 novel *BRAF* mutations which would need further functional characterization to understand their role in cancer development and treatment response.

In addition to the distinct kinase activities and inhibitor response among the *BRAF* mutations, the co-occurrence of oncogenic mutations could also affect therapeutic responses and prognosis of patients. Previous reports have demonstrated the mutual exclusivity of *BRAF* V600E with other oncogenic driver mutations [21], whereas class 2 and 3 mutations frequently co-occurred with *KRAS* mutations [16, 17]. Consistently, our analysis revealed that class 1 mutations were mutually exclusive with *KRAS* mutations (*P *< 0.01); while concurrent *KRAS* mutations were more likely to be detected in patients with class 2 and 3 mutations (class 1 vs. 2 *P *= 0.025; 1 vs. 3 *P *< 0.01). Moreover, in agreement with previous reports [8, 18], our data revealed that class 1 V600E mutations were predominant in female NSCLC patients (class 1 vs. 2 *P *= 0.008; 1 vs. 3 *P *= 0.017). However, when all the *BRAF* mutations including the non-class 1–3 mutations were collectively analyzed, *BRAF* mutations were more likely to be detected among males (*P *< 0.01). These observations between the gender distribution and *BRAF* mutation class were in contrast to the lack of gender preference of *BRAF* mutation classes reported for Caucasian NSCLC patients [16].

*BRAF* mutations have been implicated as one of the bypass mechanisms in the development of acquired resistance to epidermal growth factor receptor (EGFR) inhibitors [28]. Hence, we have excluded not only the BRAF inhibitor-treated, but also the EGFR inhibitor-treated patients in the survival analysis and confined our analysis to include only the *BRAF*-mutant advanced-stage NSCLC patients who received chemotherapy as first-line treatment regimen. Our analysis revealed comparable survival outcomes among the *BRAF* mutation classes. A study by Dagogo-Jack et al. has reported a significantly shorter overall survival for *BRAF*-mutant NSCLC patients with class 2 and 3 as compared to class 1 treated with first-line chemotherapy (2 vs. 1 *P *< 0.001; 3 vs. 1 *P *= 0.023) [16]. However, overall survival was similar for all the classes when analysis only included the patients with extra-thoracic metastases who had not received targeted therapies, indicating that the class 1 patients included in their cohort had greater proportion of thoracic metastases and their results might also have been affected by the use of targeted therapy [16]. The heterogeneity of chemotherapy regimen and metastatic sites among the patients in our cohort might have contributed to our observations on the survival outcomes. Another possibility could be the presence of concurrent mutations in oncogenic or tumor suppressor genes which still do not have definitive targeted therapy that could affect treatment response in *BRAF*-mutant patients; however, this was not included in our analysis since most patients were only sequenced with the 8-gene panel. Despite the inclusion of a large cohort in our study, our analysis is severely limited by the retrospective nature of our study. Well-designed prospective studies are needed to confirm these results.

In conclusion, *BRAF* has an overall mutation rate of 2.8% among Chinese NSCLC patients. Class 1 mutations were more likely to be detected in female patients. Class 2 and 3 mutations were more likely to have concurrent *KRAS* mutations. Our findings highlight the distinct biological characteristics of *BRAF*-mutant tumors and emphasize the need to develop more effective therapeutic strategies to improve the prognosis for these patients.

## Supplementary information

**Additional file 1: Figure S1.** *BRAF* mutations categorized as class 1 to 3 (A) and non-class 1–3 or others (B) detected in the cohort. Colored boxes depict the different functional domains along the gene. Small colored circles denote the type of mutation while the location of the circle specifies the mutation site. A patient is represented by a circle. The length of the lollipop represents the number of patients harboring the mutation. **Figure S2.** Illustration of the *in cis* configuration of the compound *BRAF* mutations as visualized using the Integrative Genomics Viewer. Alignment of sequencing reads illustrates the co-occurrence of both nucleotide substitutions on the same reads, indicating an *in cis* configuration of A. c.1406G>C (p.G469A) and c.1351G>C (p.E451Q); B. c.1514T>A (p.L505H) and c.1455G>T (p.L485F); and C. c.950C>G (S317C) and c.947C>T (p.S316L). Each gray row represents the sequencing read from a DNA fragment. Bottom bar shows the protein sequence annotation of BRAF.

**Additional file 2.** Additional tables.

## Data Availability

The dataset used and analyzed within the current study is available from the corresponding author upon reasonable request.

## Abbreviation

NSCLC: non-small cell lung cancer

## References

[1] The Cancer Genome Atlas Research N, Hammerman PS, Lawrence MS, Voet D, Jing R, Cibulskis K, et al. Comprehensive genomic characterization of squamous cell lung cancers. Nature. 2012;489: 519. 10.1038/nature11404https://www.nature.com/articles/nature11404#supplementary-information. 10.1038/nature11404 PMC3466113 22960745

[2] The Cancer Genome Atlas Research N, Collisson EA, Campbell JD, Brooks AN, Berger AH, Lee W, et al. Comprehensive molecular profiling of lung adenocarcinoma. Nature. 2014;511:543. 10.1038/nature13385https://www.nature.com/articles/nature13385#supplementary-information. 10.1038/nature13385 PMC4231481 25079552

[3] Davies H, Bignell GR, Cox C, Stephens P, Edkins S, Clegg S (2002). Mutations of the BRAF gene in human cancer. Nature.

[4] Dhillon AS, Hagan S, Rath O, Kolch W (2007). MAP kinase signalling pathways in cancer. Oncogene.

[5] Ji H, Wang Z, Perera SA, Li D, Liang MC, Zaghlul S (2007). Mutations in BRAF and KRAS converge on activation of the mitogen-activated protein kinase pathway in lung cancer mouse models. Can Res.

[6] Wan PT, Garnett MJ, Roe SM, Lee S, Niculescu-Duvaz D, Good VM (2004). Mechanism of activation of the RAF-ERK signaling pathway by oncogenic mutations of B-RAF. Cell.

[7] Yao Z, Yaeger R, Rodrik-Outmezguine VS, Tao A, Torres NM, Chang MT (2017). Tumours with class 3 BRAF mutants are sensitive to the inhibition of activated RAS. Nature.

[8] Marchetti A, Felicioni L, Malatesta S, Grazia Sciarrotta M, Guetti L, Chella A (2011). Clinical features and outcome of patients with non-small-cell lung cancer harboring BRAF mutations. J Clin Oncol.

[9] Cardarella S, Ogino A, Nishino M, Butaney M, Shen J, Lydon C (2013). Clinical, pathologic, and biologic features associated with BRAF mutations in non-small cell lung cancer. Clin Cancer Res.

[10] Joshi M, Rice SJ, Liu X, Miller B, Belani CP (2015). Trametinib with or without vemurafenib in BRAF mutated non-small cell lung cancer. PLoS ONE.

[11] Hyman DM, Puzanov I, Subbiah V, Faris JE, Chau I, Blay J-Y (2015). Vemurafenib in multiple nonmelanoma cancers with BRAF V600 mutations. N Engl J Med.

[12] Planchard D, Kim TM, Mazieres J, Quoix E, Riely G, Barlesi F (2016). Dabrafenib in patients with BRAF(V600E)-positive advanced non-small-cell lung cancer: a single-arm, multicentre, open-label, phase 2 trial. Lancet Oncol.

[13] Planchard D, Besse B, Groen HJM, Souquet PJ, Quoix E, Baik CS (2016). Dabrafenib plus trametinib in patients with previously treated BRAF(V600E)-mutant metastatic non-small cell lung cancer: an open-label, multicentre phase 2 trial. Lancet Oncol.

[14] Planchard D, Smit EF, Groen HJM, Mazieres J, Besse B, Helland A (2017). Dabrafenib plus trametinib in patients with previously untreated BRAF(V600E)-mutant metastatic non-small-cell lung cancer: an open-label, phase 2 trial. Lancet Oncol.

[15] Ding X, Zhang Z, Jiang T, Li X, Zhao C, Su B (2017). Clinicopathologic characteristics and outcomes of Chinese patients with non-small-cell lung cancer and BRAF mutation. Cancer Med.

[16] Dagogo-Jack I, Martinez P, Yeap BY, Ambrogio C, Ferris LA, Lydon C (2019). Impact of BRAF mutation class on disease characteristics and clinical outcomes in BRAF-mutant lung cancer. Clin Cancer Res.

[17] Tissot C, Couraud S, Tanguy R, Bringuier P-P, Girard N, Souquet P-J (2016). Clinical characteristics and outcome of patients with lung cancer harboring BRAF mutations. Lung Cancer (Amsterdam, Netherlands)..

[18] Li Z, Jiang L, Bai H, Wang Z, Zhao J, Duan J (2015). Prevalence and clinical significance of BRAF V600E in Chinese patients with lung adenocarcinoma. Thorac Cancer..

[19] Shan L, Qiu T, Ling Y, Guo L, Zheng B, Wang B (2015). Prevalence and clinicopathological characteristics of HER2 and BRAF mutation in chinese patients with lung adenocarcinoma. PLoS ONE.

[20] Carter J, Tseng L-H, Zheng G, Dudley J, Illei P, Gocke CD (2015). Non-pV600E BRAF mutations are common using a more sensitive and broad detection tool. Am J Clin Pathol..

[21] Kris MG, Johnson BE, Berry LD (2014). Using multiplexed assays of oncogenic drivers in lung cancers to select targeted drugs. JAMA.

[22] Villaruz LC, Socinski MA, Abberbock S, Berry LD, Johnson BE, Kwiatkowski DJ (2015). Clinicopathologic features and outcomes of patients with lung adenocarcinomas harboring BRAF mutations in the Lung Cancer Mutation Consortium. Cancer.

[23] Barlesi F, Mazieres J, Merlio JP, Debieuvre D, Mosser J, Lena H (2016). Routine molecular profiling of patients with advanced non-small-cell lung cancer: results of a 1-year nationwide programme of the French Cooperative Thoracic Intergroup (IFCT). Lancet (London, England)..

[24] Pan Y, Zhang Y, Li Y, Hu H, Wang L, Li H (2014). ALK, ROS1 and RET fusions in 1139 lung adenocarcinomas: a comprehensive study of common and fusion pattern-specific clinicopathologic, histologic and cytologic features. Lung Cancer (Amsterdam, Netherlands)..

[25] Zhou C (2014). Lung cancer molecular epidemiology in China: recent trends. Transl Lung Cancer Res.

[26] AACR Project GENIE (2017). Powering precision medicine through an international consortium. Cancer Discov.

[27] Dankner M, Rose AAN, Rajkumar S, Siegel PM, Watson IR (2018). Classifying BRAF alterations in cancer: new rational therapeutic strategies for actionable mutations. Oncogene.

[28] Ho C-C, Liao W-Y, Lin C-A, Shih J-Y, Yu C-J, Chih-Hsin Yang J (2017). Acquired BRAF V600E mutation as resistant mechanism after treatment with osimertinib. J Thorac Oncol.

