# Supplementary material for: The association between BRAF mutation class and clinical features in BRAF-mutant Chinese non-small cell lung cancer patients
Source: J Transl Med. 2019 Aug 30;17:298. doi: 10.1186/s12967-019-2036-7 (PMC6716889; doi:10.1186/s12967-019-2036-7)
Supplement: Supplementary file 1 — Additional file 1: Figure S1. BRAF mutations categorized as class 1 to 3 (A) and non-class 1–3 or others (B) detected in the cohort. Colored boxes depict the different functional domains along the gene. Small colored circles denote the type of mutation while the location of the circle specifies the mutation site. A patient is represented by a circle. The length of the lollipop represents the number of patients harboring the mutation. Figure S2. Illustration of the in cis configuration of the compound BRAF mutations as visualized using the Integrative Genomics Viewer. Alignment of sequencing reads illustrates the co-occurrence of both nucleotide substitutions on the same reads, indicating an in cis configuration of A. c.1406G>C (p.G469A) and c.1351G>C (p.E451Q); B. c.1514T>A (p.L505H) and c.1455G>T (p.L485F); and C. c.950C>G (S317C) and c.947C>T (p.S316L). Each gray row represents the sequencing read from a DNA fragment. Bottom bar shows the protein sequence annotation of BRAF. [file 12967_2019_2036_MOESM1_ESM.pptx]

## Slide 1
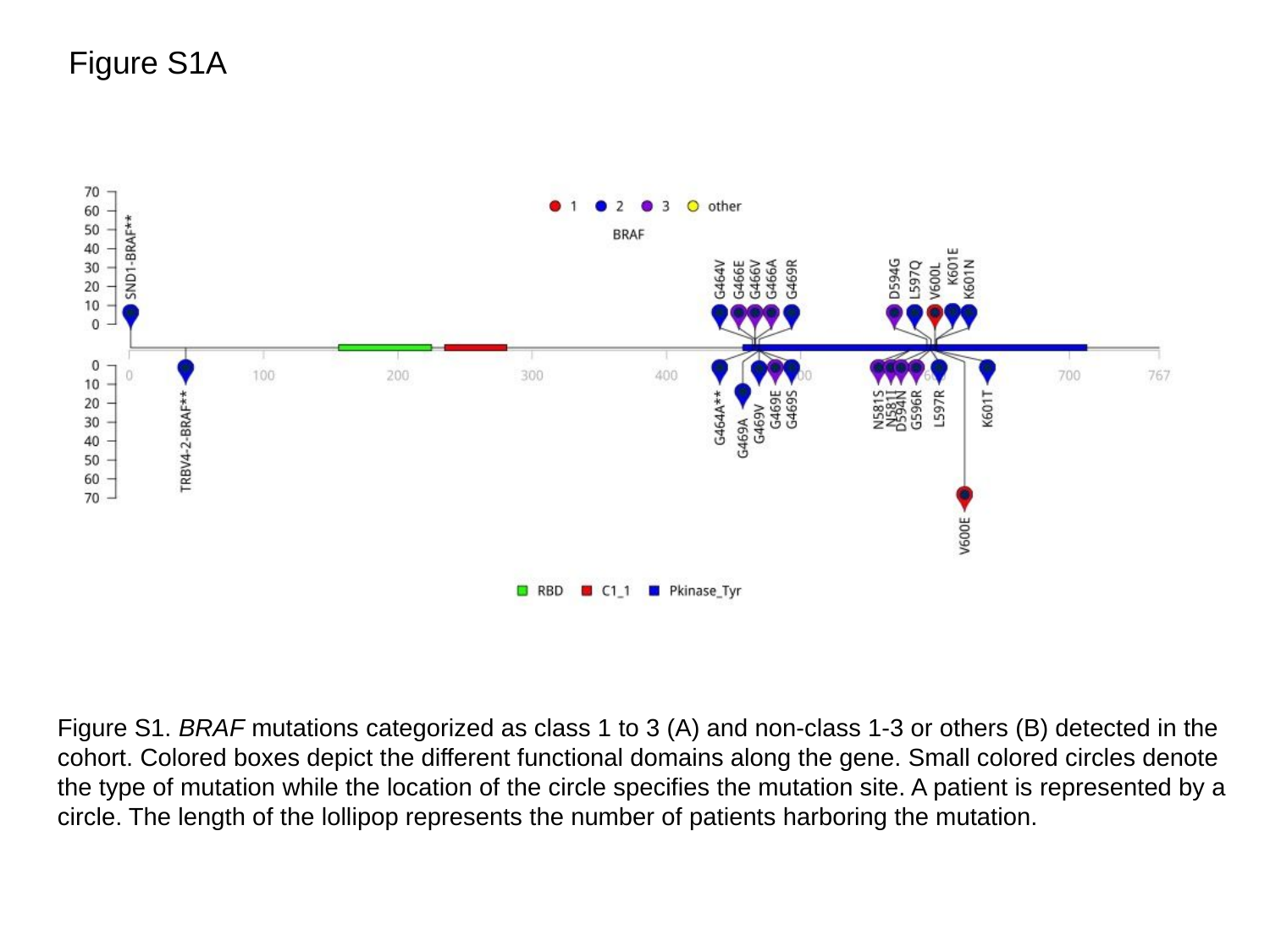

Figure S1A
Figure S1. BRAF mutations categorized as class 1 to 3 (A) and non-class 1-3 or others (B) detected in the cohort. Colored boxes depict the different functional domains along the gene. Small colored circles denote the type of mutation while the location of the circle specifies the mutation site. A patient is represented by a circle. The length of the lollipop represents the number of patients harboring the mutation.

## Slide 2
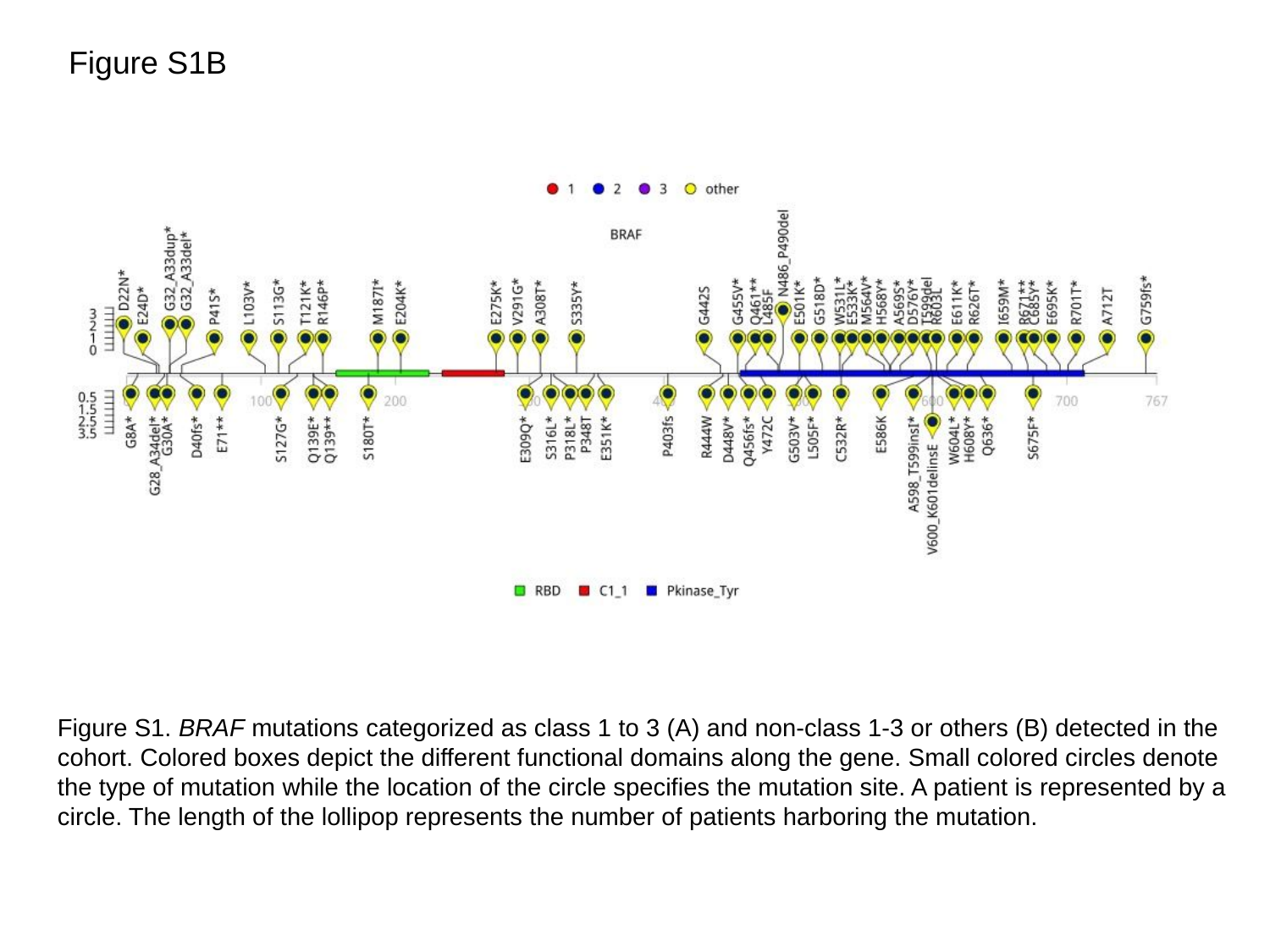

Figure S1B
Figure S1. BRAF mutations categorized as class 1 to 3 (A) and non-class 1-3 or others (B) detected in the cohort. Colored boxes depict the different functional domains along the gene. Small colored circles denote the type of mutation while the location of the circle specifies the mutation site. A patient is represented by a circle. The length of the lollipop represents the number of patients harboring the mutation.

## Slide 3
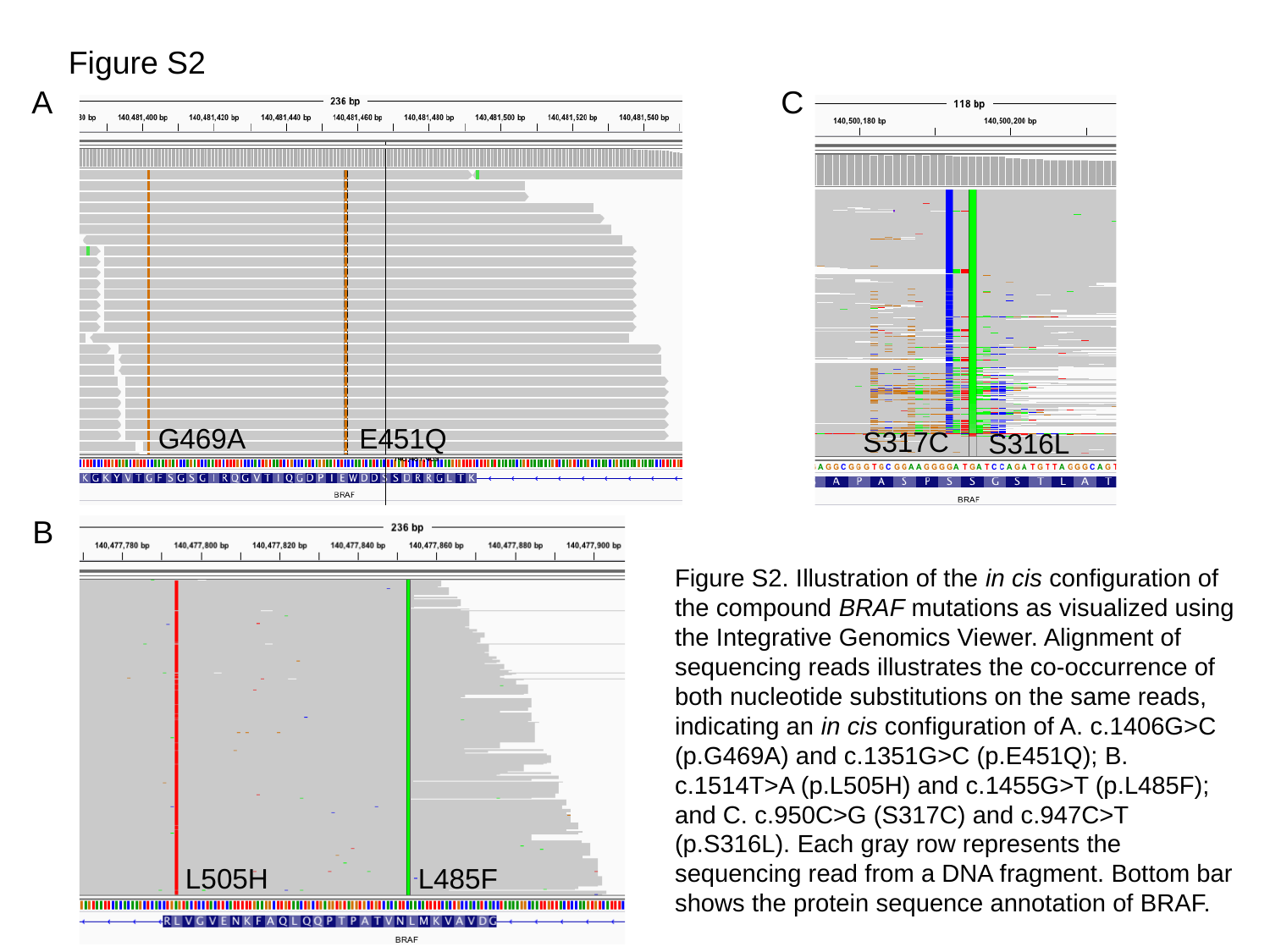

Figure S2
A
C
G469A
E451Q
S317C
S316L
B
Figure S2. Illustration of the in cis configuration of the compound BRAF mutations as visualized using the Integrative Genomics Viewer. Alignment of sequencing reads illustrates the co-occurrence of both nucleotide substitutions on the same reads, indicating an in cis configuration of A. c.1406G>C (p.G469A) and c.1351G>C (p.E451Q); B. c.1514T>A (p.L505H) and c.1455G>T (p.L485F); and C. c.950C>G (S317C) and c.947C>T (p.S316L). Each gray row represents the sequencing read from a DNA fragment. Bottom bar shows the protein sequence annotation of BRAF.
L505H
L485F
