# Supplementary material for: The association between BRAF mutation class and clinical features in BRAF-mutant Chinese non-small cell lung cancer patients
Source: J Transl Med. 2019 Aug 30;17:298. doi: 10.1186/s12967-019-2036-7 (PMC6716889; doi:10.1186/s12967-019-2036-7)
Supplement: Supplementary file 2 — Additional file 2. Additional tables. [file 12967_2019_2036_MOESM2_ESM.docx]

Table S1. List of 66 novel *BRAF* mutations detected in the cohort

| *BRAF* mutation class | Mutation type | Mutation  (nucleotide change) | Mutation  (amino acid change) | Detection count |
| --- | --- | --- | --- | --- |
| Class 2 | Missense variant | c.1391G>C | p.G464A | 1 |
| Class 2 | Fusion | Intron10_Intron8 | SND1-BRAF | 1 |
| Class 2 | Fusion | Intergenic_Intron4 | TRBV4-2-BRAF | 1 |
| Non-1-3 | Missense variant | c.23G>C | p.G8A | 1 |
| Non-1-3 | Missense variant | c.64G>A | p.D22N | 2 |
| Non-1-3 | Missense variant | c.72G>T | p.E24D | 1 |
| Non-1-3 | Disruptive inframe deletion | c.81_101del | p.G28_A34del | 1 |
| Non-1-3 | Missense variant | c.89G>C | p.G30A | 1 |
| Non-1-3 | Disruptive inframe deletion | c.95_100del | p.G32_A33del | 2 |
| Non-1-3 | Inframe insertion | c.95_100dup | p.G32_A33dup | 2 |
| Non-1-3 | Frameshift variant | c.118del | p.D40fs | 1 |
| Non-1-3 | Missense variant | c.121C>T | p.P41S | 1 |
| Non-1-3 | Splice region variant | c.138+8C>G | c.138+8C>G | 1 |
| Non-1-3 | Missense variant | c.198C>A | p.D66E | 1 |
| Non-1-3 | Stop gained | c.211G>T | p.E71* | 1 |
| Non-1-3 | Missense variant | c.307C>G | p.L103V | 1 |
| Non-1-3 | Missense variant | c.337A>G | p.S113G | 1 |
| Non-1-3 | Missense variant | c.362C>A | p.T121K | 1 |
| Non-1-3 | Missense variant | c.379A>G | p.S127G | 1 |
| Non-1-3 | Missense variant | c.415C>G | p.Q139E | 1 |
| Non-1-3 | Stop gained | c.415C>T | p.Q139* | 1 |
| Non-1-3 | Missense variant | c.437G>C | p.R146P | 1 |
| Non-1-3 | Missense variant | c.539G>C | p.S180T | 1 |
| Non-1-3 | Missense variant | c.561G>C | p.M187I | 1 |
| Non-1-3 | Splice region variant | c.610G>A | p.E204K | 1 |
| Non-1-3 | Missense variant | c.823G>A | p.E275K | 1 |
| Non-1-3 | Missense variant | c.872T>G | p.V291G | 1 |
| Non-1-3 | Missense variant | c.922G>A | p.A308T | 1 |
| Non-1-3 | Missense variant | c.925G>C | p.E309Q | 1 |
| Non-1-3 | Missense variant | c.947C>T | p.S316L | 1 |
| Non-1-3 | Missense variant | c.950C>G | p.S317C | 1 |
| Non-1-3 | Missense variant | c.953C>T | p.P318L | 1 |
| Non-1-3 | Missense variant | c.1004C>A | p.S335Y | 1 |
| Non-1-3 | Missense variant | c.1051G>A | p.E351K | 1 |
| Non-1-3 | Missense variant | c.1343A>T | p.D448V | 1 |
| Non-1-3 | Missense variant | c.1351G>C | p.E451Q | 1 |
| Non-1-3 | Missense variant | c.1364G>T | p.G455V | 1 |
| Non-1-3 | Frameshift variant | c.1365del | p.Q456fs | 1 |

Table S1. (continued)

| *BRAF* mutation class | Mutation type | Mutation  (nucleotide change) | Mutation  (amino acid change) | Detection count |
| --- | --- | --- | --- | --- |
| Non-1-3 | Stop gained | c.1381C>T | p.Q461* | 1 |
| Non-1-3 | Splice region variant | c.1433-9G>A | c.1433-9G>A | 1 |
| Non-1-3 | Missense variant | c.1501G>A | p.E501K | 1 |
| Non-1-3 | Missense variant | c.1508G>T | p.G503V | 1 |
| Non-1-3 | Missense variant | c.1513C>T | p.L505F | 1 |
| Non-1-3 | Missense variant | c.1553G>A | p.G518D | 1 |
| Non-1-3 | Missense variant | c.1592G>T | p.W531L | 1 |
| Non-1-3 | Missense variant | c.1594T>C | p.C532R | 1 |
| Non-1-3 | Missense variant | c.1597G>A | p.E533K | 1 |
| Non-1-3 | Missense variant | c.1690A>G | p.M564V | 1 |
| Non-1-3 | Missense variant | c.1702C>T | p.H568Y | 1 |
| Non-1-3 | Missense variant | c.1705G>T | p.A569S | 1 |
| Non-1-3 | Missense variant | c.1726G>T | p.D576Y | 1 |
| Non-1-3 | Disruptive inframe insertion | c.1794delinsAATC | p.A598_T599insI | 1 |
| Non-1-3 | Missense variant | c.1811G>T | p.W604L | 1 |
| Non-1-3 | Missense variant | c.1822C>T | p.H608Y | 1 |
| Non-1-3 | Missense variant | c.1831G>A | p.E611K | 1 |
| Non-1-3 | Missense variant | c.1877G>C | p.R626T | 1 |
| Non-1-3 | Missense variant | c.1977C>G | p.I659M | 1 |
| Non-1-3 | Splice acceptor variant | c.1993-1G>T | c.1993-1G>T | 1 |
| Non-1-3 | Stop gained | c.2011C>T | p.R671* | 1 |
| Non-1-3 | Missense variant | c.2024C>T | p.S675F | 1 |
| Non-1-3 | Missense variant | c.2054G>A | p.C685Y | 1 |
| Non-1-3 | Missense variant | c.2083G>A | p.E695K | 1 |
| Non-1-3 | Missense variant | c.2102G>C | p.R701T | 1 |
| Non-1-3 | Frameshift variant | c.2276del | p.G759fs | 1 |
| Non-1-3 | Fusion | Intergenic_Exon15 | BASP1P1-BRAF | 1 |
| Non-1-3 | Copy number deletion | NA | BRAF cn_del | 2 |

Abbreviations: del, deletion; dup, duplication; fs, frameshift; ins, insertion; cn_del, copy number deletion

Table S2. Compound *BRAF* mutations detected in the cohort

| Patient ID | Histology | Gender | Age | Mutation type | Mutation  (nucleotide change) | Mutation  (amino acid change) | Mutant allele frequency | COSMIC ID | *BRAF* mutation class |
| --- | --- | --- | --- | --- | --- | --- | --- | --- | --- |
| T-309 | LUAD | Male | 56 | Missense variant | c.1799T>A | p.V600E | 63.8% | COSM476 | 1 |
|  |  |  |  | Copy number amplification | NA | BRAF cn_amp | Copy number: 3.6 | NA | Non-class 1-3 |
| P-454 | LUAD | Male | 60 | Missense variant | c.1351G>C | p.E451Q | 2.03% | NA | Non-class 1-3 |
|  |  |  |  | Missense variant | c.1406G>C | p.G469A | 2.02% | COSM460 | 2 |
| P-838 | LUADSC | Male | 61 | Missense variant | c.1803A>T | p.K601N | 23.22% | COSM6265 | 2 |
|  |  |  |  | Missense variant | c.2134G>A | p.A712T | 23.22% | COSM2861420 | Non-class 1-3 |
| T-883 | LUAD | Male | 78 | Missense variant | c.198C>A | p.D66E | 20.2% | NA | Non-class 1-3 |
|  |  |  |  | Missense variant | c.1742A>G | p.N581S | 29.1% | COSM462 | 3 |
| T-006 | LUAD | Female | 61 | Missense variant | c.947C>T | p.S316L | 19.1% | NA | Non-class 1-3 |
|  |  |  |  | Missense variant | c.950C>G | p.S317C | 12.0% | NA | Non-class 1-3 |
| P-534 | LUAD | Male | 73 | Missense variant | c.1455G>T | p.L485F | 8.79% | COSM30728 | Non-class 1-3 |
|  |  |  |  | Missense variant | c.1514T>A | p.L505H | 7.95% | COSM243226 | Non-class 1-3 |
| T-598 | LUSC | Male | 59 | Splice region variant | c.1433-9G>A | c.1433-9G>A | 20.5% | NA | Non-class 1-3 |
|  |  |  |  | Stop gained | c.2011C>T | p.R671* | 23.2% | NA | Non-class 1-3 |

Note: T and P in the Patient ID denote sample types, T, tissue; P, plasma.

NA in COSMIC ID denotes not applicable

Abbreviations: LUAD, lung adenocarcinoma; LUADSC, lung adenosquamous carcinoma; LUSC, lung squamous cell carcinoma; cn_amp, copy number amplification

Table S3. Distribution of concurrent oncogenic driver mutations detected in the cohort

| Oncogenic driver mutation | Class 1 | Class 2 | Class 3 | Non-class 1-3 | *P*-value  (1 vs. 2) | *P*-value  (1 vs. 3) | *P*-value  (I vs. 2 + 3) | *P*-value  (2 vs. 3) |
| --- | --- | --- | --- | --- | --- | --- | --- | --- |
| KRAS G12X, G13X, Q61X | 0 (0%) | 4 (7.8%) | 6 (19.3%) | 6 (7.4%) | **0.025** | **<0.01** | **0.001** | 0.16 |
| EGFR L858R, T790M, C797S, G719X, L861Q, S768I, exon 19 deletion, exon 19 insertion, exon 20 insertion | 14 (18.7%) | 8 (15.7%) | 2 (6.4%) | 25 (30.9%) | 0.8 | 0.14 | 0.27 | 0.3 |
| *ALK* fusion | 2 (2.7%) | 0 (0%) | 0 (0%) | 1 (1.2%) | 0.51 | 1 | 0.22 | 1 |
| *ROS1* fusion | 0 (0%) | 0 (0%) | 0 (0%) | 2 (2.5%) | 1 | 1 | 1 | 1 |
| *RET* fusion | 0 (0%) | 0 (0%) | 0 (0%) | 0 (0%) | 1 | 1 | 1 | 1 |
| *ERBB2* gene amplification, exon 20 insertion, S310X | 0 (0%) | 0 (0%) | 0 (0%) | 4 (4.9%) | 1 | 1 | 1 | 1 |
| *MET* amplification and exon 14 skipping | 0 (0%) | 1 (2.0%) | 1 (3.2%) | 1 (1.2%) | 0.4 | 0.29 | 0.49 | 1 |

*P*-values in **bold-face** denotes statistical significance
